# Tumor-derived exosomal circRNA_102481 contributes to EGFR-TKIs resistance via the miR-30a-5p/ROR1 axis in non-small cell lung cancer

**DOI:** 10.18632/aging.203011

**Published:** 2021-05-05

**Authors:** Bo Yang, Fei Teng, Liang Chang, Jian Wang, De-Long Liu, Yong-Sheng Cui, Guang-Hu Li

**Affiliations:** 1 Department of Thoracic Surgery, The First Hospital of Jilin University, Chaoyang, Changchun, Jilin, China; 2 Key Laboratory for Biorheological Science and Technology of Ministry of Education, Chongqing University Cancer Hospital, Shapingba, Chongqing, China; 3 Department of Neurovascular Surgery, The First Hospital of Jilin University, Chaoyang, Changchun, Jilin, China

**Keywords:** EGFR-TKIs resistance, exosomal, circRNA_102481, miR-30a-5p, ROR1

## Abstract

Exosomes are messengers for intercellular communication and signal transduction. Circular RNA (circRNA) abnormal expression and regulation are involved in the occurrence and development of a variety of tumors. In the present study, exosomes in the serum of five patients with non-small cell lung cancer (NSCLC) were isolated before and after EGFR-TKIs resistance, and the circRNA expression profile was screened using a circRNA microarray. The effects of the exosome circRNA_102481 on cell proliferation and apoptosis were analyzed. The interaction between miR-30a-5p and circRNA_102481 or ROR1 was predicted by starBase software, and was confirmed by RNA pull-down and dual-luciferase reporter assays. The results showed that exosomes containing circRNA_102481 were significantly up-regulated in NSCLC with EGFR-TKIs resistance (p<0.05), and that circRNA_102481 was mainly secreted by EGFR-TKIs resistance cell via exosomes (p<0.05). Both circRNA_102481 silencing and si-circRNA_102481 transported by exosomes could inhibit EGFR-TKIs resistance cell proliferation and promote cell apoptosis and circRNA_102481 overexpression could promote EGFR-TKIs sensitive cell proliferation and inhibit cell apoptosis *in vitro* (p<0.05). CircRNA_102481 served as a miR-30a-5p sponge to regulate ROR1 expression (p<0.05). Furthermore, the expression of circRNA_102481 in exosomes was associated with TNM stage, tumor differentiation status, brain metastasis, and PFS and OS duration. Therefore, it was concluded that tumor-derived exosomal circRNA_ 102481 could contribute to EGFR-TKIs resistance via the microRNA-30a-5p/ROR1 axis in NSCLC. Exosomal circRNA_102481 may serve as a novel diagnostic biomarker and a therapeutic target for EGFR-TKIs resistance in NSCLC.

## INTRODUCTION

Epidermal growth factor receptor tyrosine kinase inhibitors (EGFR-TKIs) were successfully treatment for NSCLC with EGFR sensitive mutation [1], such as gefitinib, erlotinib, and afatinib, but almost all of the patients for whom the initial therapy was effective will present progress after less than 8-10 months, and produce the acquired drug-resistance [2, 3]. Although the listing of third-generation EGFR-TKIs (osimertinib) has solved resistance problem of First- and second- generation EGFR-TKIs, there are still a large number of First- and second-generation resistant patients who cannot benefit from the treatment of osimertinib [4, 5].

Exosomes are small vesicles with a diameter of 40-100 nm, which are secreted by the body cells and released into the extracellular microenvironment, and are widely distributed in saliva, plasma, milk, urine and other body fluids [6]. Exosomes can derive from various cell types, and they constitute an important mechanism to control intercellular communication and signal transduction in the cytoplasm, as well as transcription in the nucleus by interactions between cells, thus determining cell proliferation, migration, growth, differentiation and death [7]. Exosomes contain biological macromolecules such as RNA, protein and nucleic acid. These molecules are wrapped and protected by the lipid membrane of exosomes, which can maintain their original stability and biological functions [8]. Exosomes can enter the external environment of cells via paracrine pathways, directly stimulate target cells by acting as signal complexes, and transport organelles, proteins, nucleic acids and membrane receptors to the target cell [9]. Exosomes affect the transcription and translation of target genes, or regulate cell signaling pathways through multiple channels to affect the function and physiological state of target cells [10]. As a major pattern to maintain the exchange of materials and communication of information among organelles, exosomes can be secreted by a variety of cells, and are closely associated with tumors [11]. Furthermore, exosomal blood-based screening (liquid biopsy) is a quick and non-invasive method of evaluation with high repeatability [12].

Circular RNA (circRNA) is a novel class of non-coding RNA (ncRNA) characterized by covalently closed loops without 3’ or 5’-end [13]. Due to its particular closed loop structure, circRNA is not easily degraded by endonuclease, and is stably expressed in various organisms [14, 15]. Compared with linear RNA, circRNA is more stable in nature, has more functions, is more conserved in evolution, and its number is much greater [16, 17]. CircRNA is currently the best biomarker and therapeutic target among ncRNAs [18, 19]. Therefore, exosomes containing circRNA have become a hot research topic. Recent studies [20, 21] showed that tumor-derived exosomal circRNA plays an important regulatory role in tumorigenesis, growth, invasion and metastasis. However, the role of tumor-derived exosomal circRNA on EGFR-TKIs resistance remains unclear.

In the present study, the profile of exosomes containing circRNA before and after EGFR-TKIs resistance was successfully established. In addition, tumor-derived exosomal circRNA_102481 was identified as a key circRNA in the EGFR-TKIs resistance process. Furthermore, our results suggested that circRNA_102481 may contribute to EGFR-TKIs resistance via the miR-30a-5p/ROR1 axis and is associated with the prognosis of NSCLC.

## MATERIALS AND METHODS

### Patients enrolled

We first collected 5 cases for exosome analysis, and then gradually increased the samples to 58 cases (Supplementary Table 1). A total of 58 NSCLC patients treated with EGFR-TKIs (gefitinib or erlotinib) were enrolled in our study from September 2017 to March 2019 (Supplementary Table 2). According to the National Comprehensive Cancer Network (NCCN) guidelines, patients with EGFR sensitive mutation and those with objective response or stable disease to EGFR-TKIs treatments were regarded as EGFR-TKIs sensitive patients. Patients who suffered progressive disease or recurrent disease within 6 months were classified as EGFR-TKIs resistance patients. Serum exosomes of 58 NSCLC patients were extracted before and after EGFR-TKIs resistance. The subject was informed and approved by medical ethics committee of the first hospital of Jilin University. Specimens were selected for the patient's 'consent in advance and informed consent forms were signed.

### Cell lines

The gefitinib sensitive cell lines PC9 and the corresponding gefitinib resistant cell lines PC9/GR, the erlotinib sensitive cell lines HCC827 and the corresponding erlotinib resistant cell lines HCC827/ER were purchased from the Cell Bank of the Chinese Academy of Sciences. The cells after resuscitation were cultured in high glucose DMEM medium, containing 10% fetal bovine serum and incubated at a constant incubator at 37° C, 5% CO_2_. All the tissues used in the experiments were informed and approved by ethics committee of the first hospital of Jilin University.

### Extraction and identification of exosomes

Exosomes in serum and medium were isolated by the ExoQuick method. Centrifuge the sample at 3,000×g for 15 minutes and take the supernatant. After filtering with 0.22-mm PVDF filter, add the precipitant, mix and shake, store in a refrigerator at 4° C for 30 minutes, and centrifuge at 1,500×g for 5 minutes. Discard the supernatant Precipitate in 100 ML PBS and place in a constant temperature refrigerator at 80° C. Exosomes were collected from the pellet and re-suspended in PBS. The exosomes morphology was observed under a transmission electron microscope (TEM). The size distribution and concentration of exosomes were analyzed by nanoparticle-tracking analysis. The exosomal-related marker protein of CD63 and TSG101 were detected by western-blotting assay.

### circRNA microarray

The exosomes RNA of five pair’s samples were extracted using RNeasy Mini Kit (Qiagen, Hilden, Germany) according to the manufacturer's protocol. Sample labeling and array hybridization were performed. Total RNAs were treated with RNase R to remove linear RNAs and quantified and quality was verified using NanoDrop® ND-1000 UV-Vis Spectrophotometer (NanoDrop, Wilmington, DE, USA). The enriched circRNAs were amplified and transcribed into cDNA. Lastly, the circRNA profile was measured using Arraystar high-throughput human circRNA microarray V2.0 (8×15 K, Arraystar, Inc.). CircRNAs with ≥2.0 fold-changes (FC) and p < 0.05 were selected as circRNAs with significant differential expression.

### Quantitative real-time PCR

The exosomes RNA of five pair’s samples were isolated with Trizol reagent kit. 5 μg of tRNA were used for reverse transcription with First Strand cDNA Synthesis Kit in qRT-PCR system. The PCR system reaction were at 95° C for 15min, followed by 40 cycles at 94° C for 15s, and 20 cycles at 55° C for 30s and 20 cycles at 70° C for 30s were performed. The relative expression levels were calculated by using the 22−Δ∆Ct method. ΔCt=Cttarget -CtU6. The qPCR primers were designed using Primer Premier Software (version 5; Premier Biosoft International). The forward (F) and reverse (R) sequence of hsa_circRNA_102481, hsa_circRNA_063313, hsa_circRNA_103827, hsa_circRNA_405296, hsa_circRNA_ 000367, hsa_circRNA_406587, miR-30a-5p, ROR1 mRNA and U6, GAPDH (control sequence) were shown in Table 1.

**Table 1:** The RT-qPCR primers sequences.

**Items**	**Sequences**	
hsa_circRNA_102481	F	5'-ACATCCACAATGGCACTGA-3'
	R	5'-AGGAGGAGATGAGTTGGAA-3'
hsa_circRNA_063313	F	5'-AAAACACTGTTGACAATAA-3'
	R	5'-TGGCTCCTACCAGAGAGCT-3'
hsa_circRNA_103827	F	5'-CATAGCAACTGAGGGCTT-3'
	R	5'-GCAACATGCCTATGATTT-3'
hsa_circRNA_405296	F	5'-CGCACACCATCATGCCCAGA-3'
	R	5'-ATCTATAGAGAAAATGGAATC-3'
hsa_circRNA_ 000367	F	5'-GTCCAGGTCCATTCCA-3'
	R	5'-TAACTGGTCCCAGTAAGCA-3'
hsa_circRNA_406587	F	5'-TGCCAAGTCAGGGGAGAGG-3'
	R	5'-TCCTTGAACTCCACTGATACTT-3'
hsa_miR-30a-5p	F	5'-GAAGGUCAGCUCCUACAAAUGU-3'
	R	5'-UUAGUCAGAGUAACGAAAUAUU-3'
ROR1 mRNA	F	5'-TGCCAGCCCAGTGAGTAATCT-3'
	R	5'-GCCAATGAAACCAGCAATCTG-3'

### Cell transfection

Small interfering RNA (siRNA/si) targeting circRNA_102481 (si-circRNA_102481) and corresponding control (si-NC) labeled with a green fluorescent protein (GFP), miR-30a-5p mimics, miR-30a-5p inhibitor and its matched control (miR-30a-5p NC) were transfected using Lipofectamine^TM3000^ reagent (Invitrogen Co. Ltd, USA) according to the manufacturer’s instruction. The transfection efficiency was observed under a confocal laser scanning microscope and verified using RT-qPCR after 48 h transfection.

### MTT assay

After 48 h transfection, cells in logarithmic growth phase were seeded into 96-well culture dish and cultured with different doses gefitinib (0.0, 0.25, 0.5, 1.0, 2.0, 4.0, 8.0, 16.0 μmol/L) or erlotinib (0.0, 0.5, 1.0, 2.0, 4.0, 8.0, 16.0, 32.0 μmol/L) in the 37° C, 5% CO_2_ incubator. After cultured for 48 h, A549/PTX and H292/PTX cells in each group were incubated with 20 μl of 5.0 mg/ml 3-(4,5- Dimethylthiazol-2-yl)-2,5- diphenyltetrazolium bromide (MTT) for 4 h and 100 μl of dimethyl sulfoxide (DMSO) for 30 min. The absorbance (OD) at 490 nm was measured by a micro plate reader.

### Annexin V-FITC/PI assay

After 48 h transfection, cells in logarithmic growth phase were seeded into 6-well culture dish and cultured with 2μmol/L gefitinib or 4μmol/L erlotinib in the 37° C, 5% CO_2_ incubator. After cultured for 48 h, cells in each group were subsequently incubated with 5 μl Annexin V combined Fluorescein isothiocyanate (FITC) and 5 μl Propidium Iodide (PI) solutions at 37° C for 20 min in the dark. The apoptotic cells were measured using flow cytometer (BD Biosciences Co. Ltd, USA).

### Dual-luciferase reporter assay

The firefly luciferase reporter plasmid psiCHECK2 with circRNA_102481-3’UTR or ROR1-3’UTR containing the wide type sequences (wt) or mutant sequences (mut) were co-transfected with miR-30a-5p mimic or miR-30a-5p NC into PC9/GR and HCC827/ER cells using Lipofectamine ^TM3000^ reagent for 48h. The Firefly and Renilla luciferase activity was detected by a Dual-Luciferase Reporter Assay System, with Renilla luciferase as normalized control.

### Western blot

After 48 h transfection, cells in logarithmic growth phase were digested and total proteins were collected. 50μg isolated protein were separated by 10% polyacrylamide gel electrophoresis and transferred to PVDF membrane, and blocked with 5% non-fat milk for 1 h. Then, the membranes were incubated with primary Anti-ROR1 antibody (1:1000 dilution, catalogue number: ab5073, Abcam Co. Ltd, USA) for 4 h at 37° C and followed by horseradish peroxidase (HRP)-conjugated secondary antibodies. Protein bands were visualized using the Pierce enhanced chemiluminescent (ECL) system.

### Statistical analysis

GraphPad Prism 8.1 (GraphPad Software Company, San Diego, CA, USA) and SPSS 18.0 statistical software (SPSS Inc., Chicago, IL, USA) was used for the statistical analysis.. The data are expressed as the mean ± SD. Statistical differences between two groups were determined using an unpaired Student’s t-test or an χ^2^-test, whereas statistical differences between >2 groups were analyzed using a one-way analysis of variance, followed by Tukey's multiple comparison test. Fisher exact and χ2 tests were used to compare categorical variables. Continuous variables were compared using the Wilcoxon rank-sum test or Kruskal-Wallis test. OS durations between groups were compared using the Kaplan-Meier method and log-rank test. *p*<0.05 was considered statistically significant difference.

## RESULTS

### Identification of exosomes

**Figure 1:**
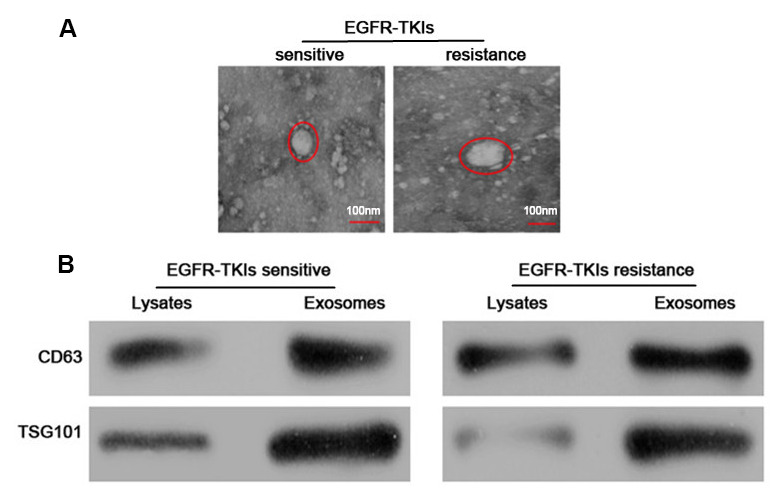
**Identification and characterization of serum exosomes.** (**A**) Representative electron micrographs of serum exosomes obtained by TEM analysis (right, bars=100 nm). (**B**) Western blots analysis of exosomal markers, including CD63 and TSG101 in exosomes and cell lysates.

The serum exosomes of five paired samples were isolated by the ExoQuick method. The exosomes morphology was observed under a transmission electron microscope, which revealed that the exosomes were regular, and the majority of them round, elliptical or disc-shaped vesicles with a uniform size. In addition, a lipid membrane was observed around the exosomes (Figure 1A). Western blot analysis confirmed that exosomal markers, including CD63 and TSG101 proteins, were highly expressed in exosomes (Figure 1B). These results indicated that exosomes were successfully isolated from samples’ serum.

### Successful establishment of exosomal circRNA expression profile

The exosomes RNA of five pair’s samples was extracted using RNeasy Mini Kit (Qiagen GmbH) and the circRNA profile was measured using Arraystar high-throughput human circRNA microarray V2.0 (8x15 K; Arraystar, Inc.). Scatter plot Supplementary Figure 1A was used for assessing the variation or reproducibility of circRNA expression. The values of the x and y axes in the scatter plot correspond to the normalized signals of the samples (log2 scaled). Volcano plots Supplementary Figure 1B were constructed using fold-change and P-values for visualizing differential expression between two different conditions. The volcano plots and scatter plots showed that the differences in circRNA expression were variable.

### circRNA_102481 is significantly up-regulated in serum exosomes of patients with EGFR-TKIs resistance

**Figure 2:**
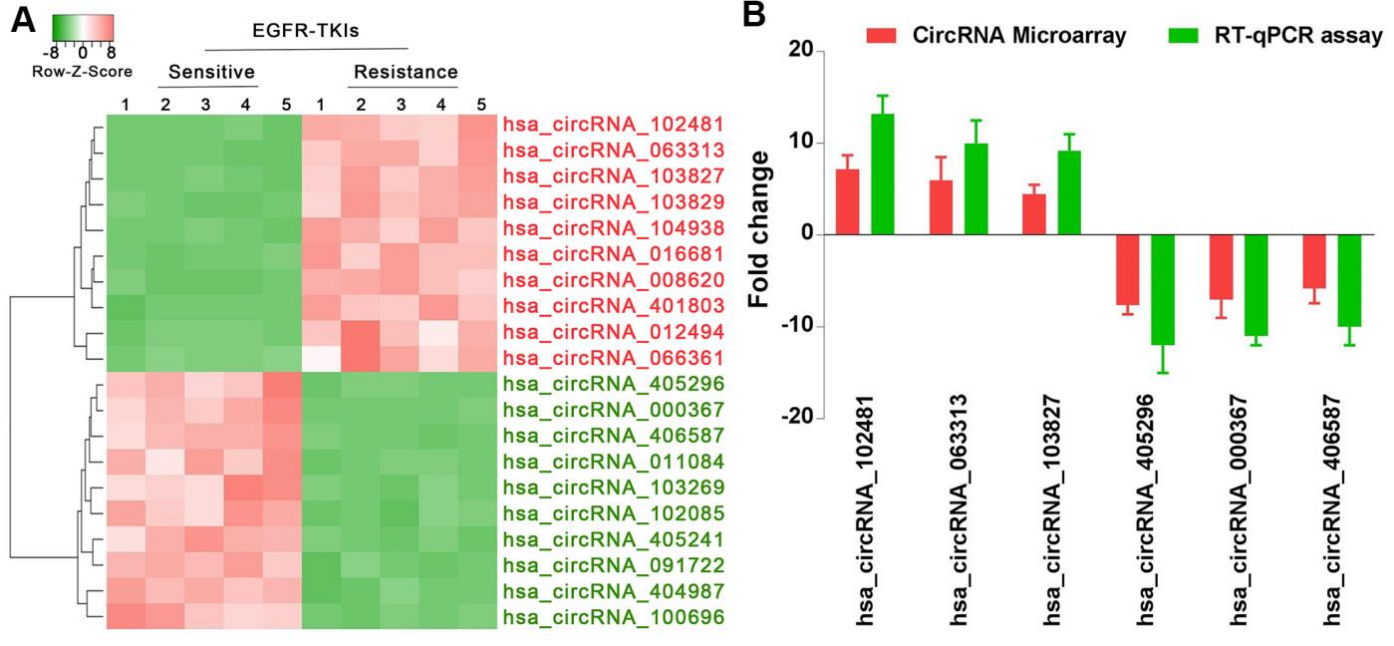
**circRNA_102481 is significantly up-regulated in serum exosomes of patients with EGFR-TKIs resistance.** (**A**) Hierarchical clustering analysis showed the top 10 most increased and increased circRNAs in serum exosomes. (**B**) RT-qPCR validation.

Through standardization of data processing and analysis, hierarchical clustering analysis (Figure 2A) showed the top 10 most increased circRNAs in serum exosomes of patients with EGFR-TKIs resistance compared with EGFR-TKIs-sensitive patients. In addition, reverse transcription-quantitative (RT-qPCR) analysis confirmed that hsa_circRNA_102481, hsa_circRNA_063313 and hsa_circRNA_103827 were significantly up-regulated, while hsa_circRNA_405296, hsa_circRNA_000367 and hsa_circRNA_406587 were obviously down-regulated in serum exosomes of EGFR-TKIs-resistant patients (Figure 2B; p<0.05). Our results illustrated that circRNA microarray analysis data were consistent with the RT-qPCR results regarding the expression levels of the aforementioned six circRNAs. Among them, circRNA_102481 showed the highest up-regulation (it was significantly up-regulated by 7.2 fold) in serum exosomes of EGFR-TKIs-resistant patients.

### Exosomal circRNA_102481 derives from EGFR-TKIs-resistant cells

**Figure 3:**
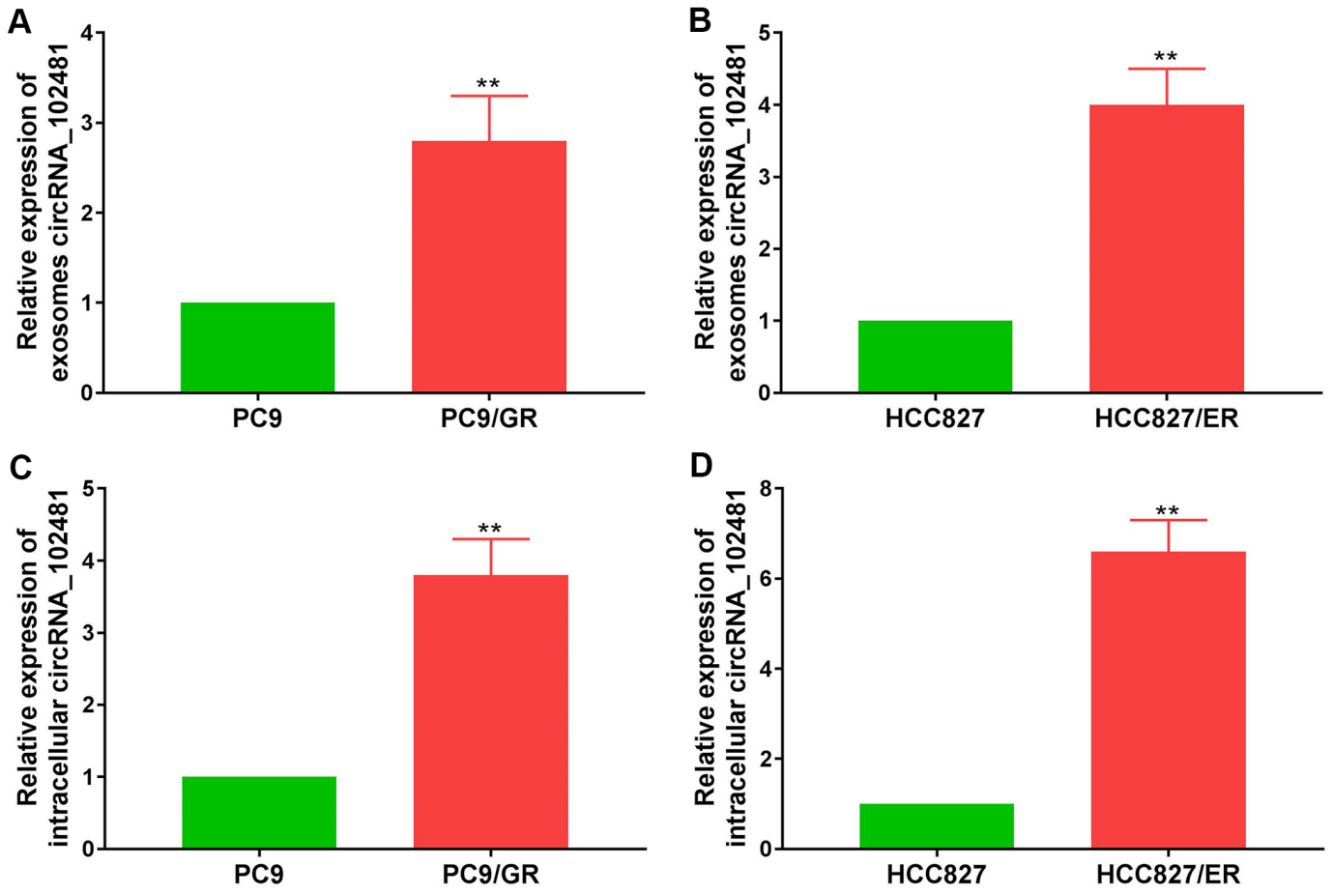
**circRNA_102481 is mainly secreted via exosomes.** (**A**, **B**) circRNA_102481 was unregulated in EGFR-TKIs resistant cells (PC9/GR and HCC827/ER), **p< 0.01 versus matched sensitive cells (PC9 and HCC827). (**C**, **D**) circRNA_102481 was significantly up-regulated in exosomes of EGFR-TKIs resistant cells (PC9/GR and HCC827/ER), **p< 0.01 versus matched sensitive cells (PC9 and HCC827).

Exosomes can derive from various types of cells. In order to demonstrate whether exosomal circRNA_102481 derived from EGFR-TKIs-resistant cells, the EGFR-TKIs-resistant PC9/GR and HCC827/ER cell lines were selected as cell models for further verification. RT-qPCR assay showed that circRNA_102481 was up-regulated in EGFR-TKIs resistant cells (PC9/GR and HCC827/ER) compared with its expression level in sensitive cells (PC9 and HCC827) (Figure 3A, 3B; p<0.05). Exosomes in cell culture medium (CCM) were isolated by the ExoQuick method, and the total RNA of exosomes was extracted using the RNeasy Mini Kit. RT-qPCR assay showed that circRNA_102481 was significantly up-regulated in exosomes of EGFR-TKIs resistant cells (PC9/GR and HCC827/ER) compared with its expression level in sensitive cells (PC9 and HCC827) (Figure 3C, 3D; p<0.05).

**Figure 4:**
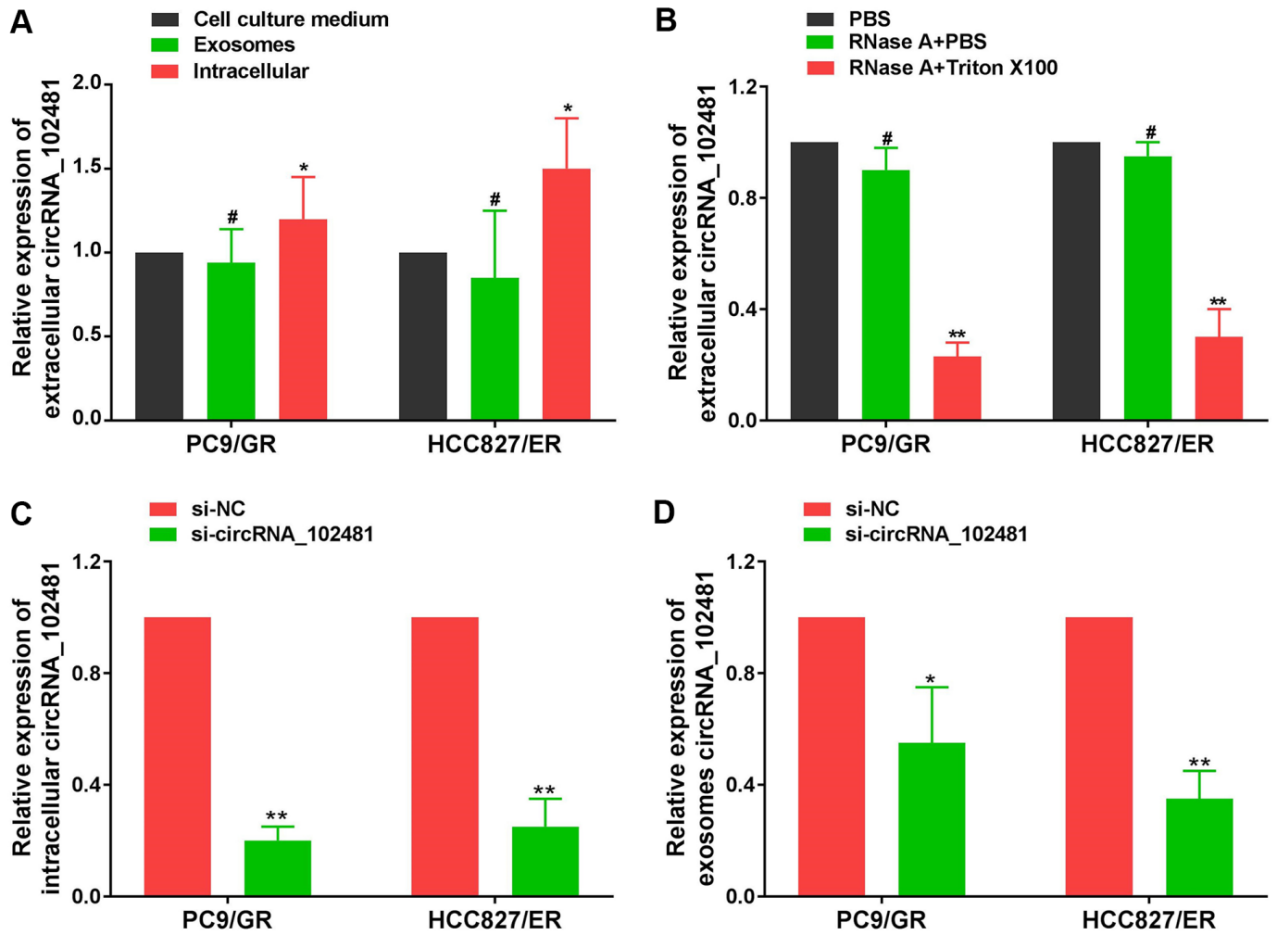
**Exosomal circRNA_102481 derived from EGFR-TKIs resistance cells.** (**A**) circRNA_102481 expression levels in exosomes were almost equal to those in CCM, but, the levels of circRNA_102481 expression in EGFR-TKIs-resistant cells were significantly higher than in CCM and exosomes, ^#^*p* > 0.05, *p< 0.05. (**B**) RT-qPCR was performed to detect the expression level of circRNA_102481 after treatment with 1 μg/ml RNase alone or combined with 0.1% Triton X-100 for 30 min, ^#^*p* > 0.05, *p< 0.05 compared to the PBS control. (**C**) Transfection efficiency of circRNA_102481 was verified by a confocal laser scanning microscope and RT-qPCR assay. **P < 0.01 versus si-NC group cells. (**D**) circRNA_102481 was significantly down- regulated in exosomes of si-circRNA_ 102481 cells. *p<0.05, **p<0.01 versus si-NC group exosomes. CCM: cell culture medium.

The total RNA of CCM in each cell line was also extracted using the RNeasy Mini Kit. RT-qPCR assay demonstrated that the circRNA_102481 expression levels in exosomes were almost equal to those in CCM, but, the levels of circRNA_102481 expression in EGFR-TKIs-resistant cells were significantly higher than in CCM and exosomes (Figure 4A; p<0.05), indicating that extracellular circRNA_102481 was contained in exosomes, which means that circRNA_102481 is mainly secreted to CCM in the form of exosomes. In order to verify whether circRNA_102481 is secreted through packaging into exosomes, the extracellular circRNA_102481 expression level after treatment with ribonucleic (RNase) was detected. As revealed in Figure 4B, circRNA_102481 in CCM was hardly influenced by treatment with RNase alone, but its level significantly decreased when upon treatment RNase and Triton X-100 (which can dissolve lipids and destroy membrane structures) simultaneously (p>0.05), suggesting that extracellular circRNA_102481 was protected by a membrane instead of being directly secreted to the extracellular medium.

In addition, si-circRNA_102481 and the corresponding control si-NC were transfected into PC9/GR and HCC827/ER cells, and the transfection efficiency was verified by a confocal laser scanning microscope and RT-qPCR assay (Figure 4C; p<0.05). Furthermore, exosomes in the CCM of the si-NC and si-circRNA_102481 groups were isolated. RT-qPCR assay showed that circRNA_102481 was significantly down-regulated in exosomes of si-circRNA_ 102481 cells compared with its level in matched si-NC cells (Figure 4D; p<0.05), which indicated that exosomes circRNA_102481 can secreted by PC9/GR and HCC827/ER cells. These results suggested that circRNA_102481 is mainly secreted by cancer cells via exosomes.

### Effects of circRNA_102481 on EGFR-TKIs-resistant NSCLC cells

**Figure 5:**
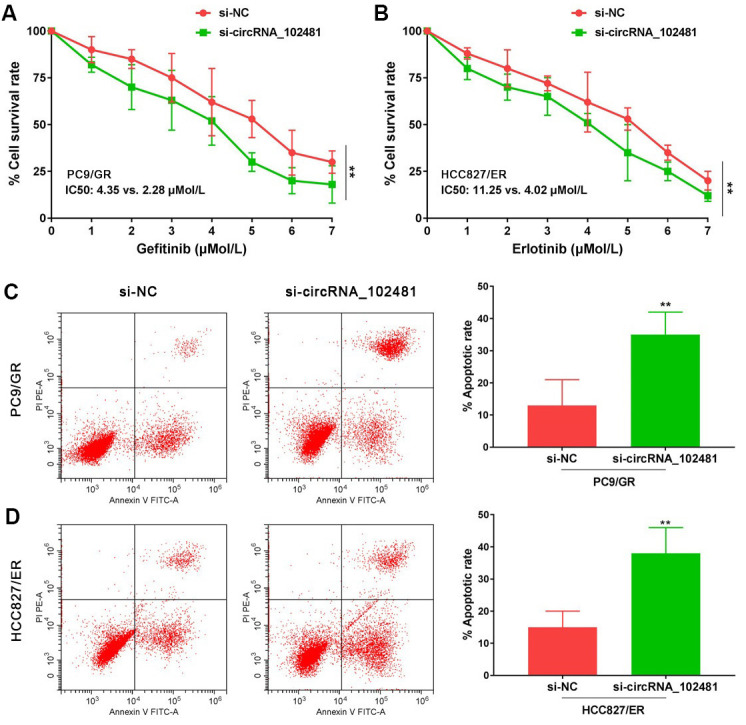
**Functional validation assay of circRNA_102481 on EGFR-TKIs resistant cells *in vitro* (n=3).** (**A**, **B**) MTT assay showed the effects of circRNA_102481 on EGFR-TKIs resistant cell proliferation. (**C**, **D**) Annexin V/PI assay showed the effects of circRNA_102481 on EGFR-TKIs resistant cell apoptosis. si-circRNA_102481: siRNA targeting circRNA_102481. **p<0.01 versus si-NC group.

In our study, si-circRNA_102481 was successfully transfected into PC9/GR and HCC827/ER cells to silence circRNA_102481 expression. MTT and Annexin V/PI assays were conducted to investigate the role of circRNA_102481 on PC9/GR and HCC827/ER cells. The MTT assay results showed that, with gefitinib treatment, the cell viability of PC9/GR cells was significantly inhibited following the interference of circRNA_102481, and the half-maximal inhibitory concentration (IC50) value of gefitinib was markedly decreased in the si-circRNA_102481 group compared with that in the si-NC group (IC50, 2.28 vs. 4.35 μmol/l; Figure 5A; p<0.05). Similarly, with erlotinib treatment, the cell viability of HCC827/ER cells was also significantly inhibited following the interference of circRNA_102481, and the IC50 value of erlotinib was markedly decreased in the si-circRNA_102481 group compared with that in the si-NC group (IC50, 4.02 vs. 11.25 μmol/l; Figure 5B; p<0.05).

In addition, after treatment with 2 μmol/l gefitinib or 4 μmol/l erlotinib for 24 h, the results of Annexin V/PI assay demonstrated that the apoptosis rate was markedly increased in the si-circRNA_102481 group compared with that in the corresponding si-NC group (Figure 5C, 5D; p<0.05). These results indicated that circRNA_ 102481 silencing could inhibit EGFR-TKIs resistance NSCLC cell proliferation and promote cell apoptosis *in vitro*, thereby enhancing EGFR-TKIs sensitivity to NSCLC.

### Effects of exosomes containing circRNA_102481 on EGFR-TKIs-resistant NSCLC cells

**Figure 6:**
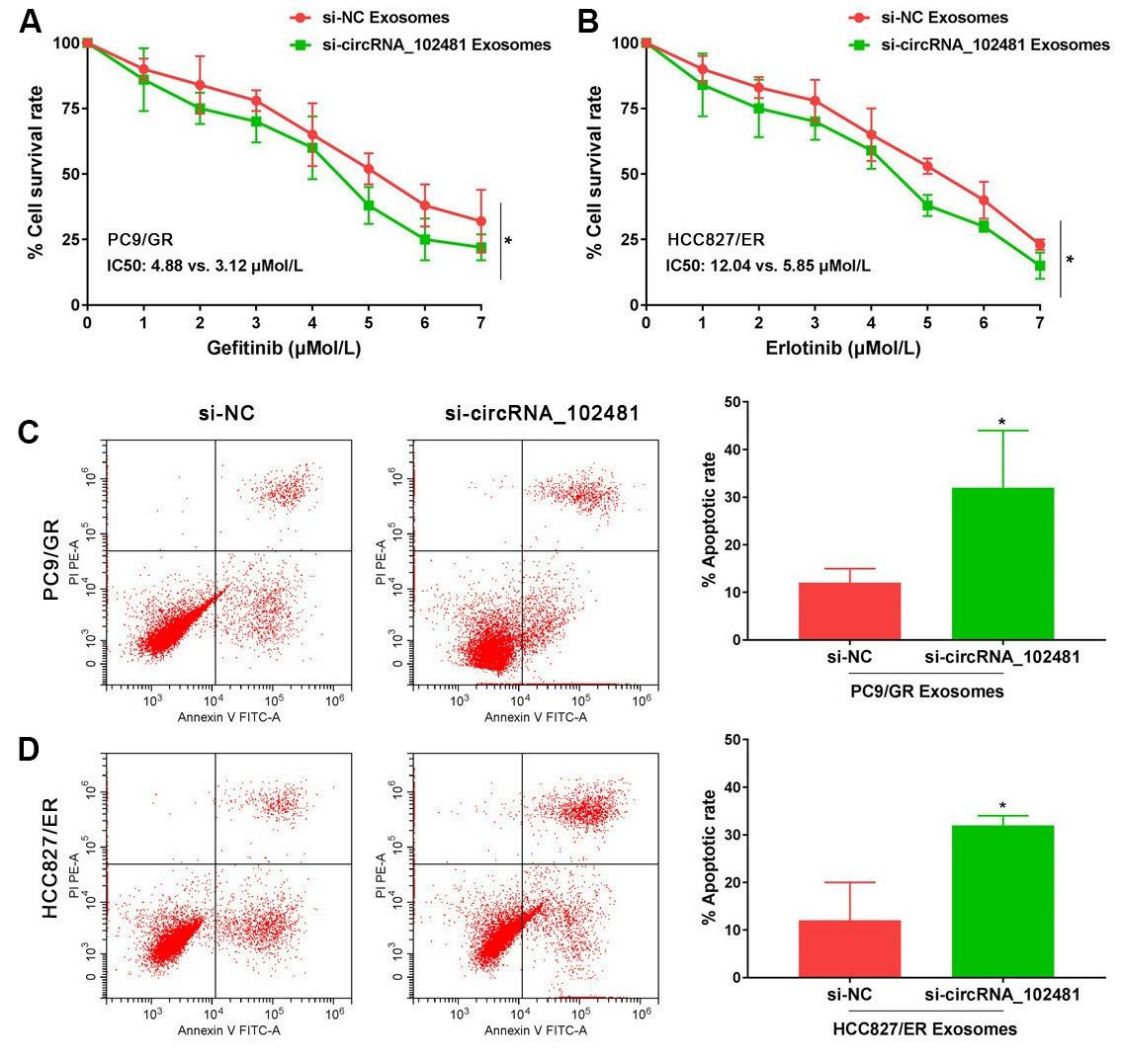
**Functional validation assay of exosomes circRNA_102481 on EGFR-TKIs resistant cells *in vitro* (n=3).**(**A**, **B**) MTT assay showed the effects of exosomes circRNA_102481 on EGFR-TKIs resistant cell proliferation. (**C**, **D**) Annexin V/PI assay showed the effects of exosomes circRNA_102481 on EGFR-TKIs resistant cell apoptosis. si-circRNA_102481: siRNA targeting circRNA_102481. *p<0.05 versus si-NC group.

In order to determine the role of exosomes-derived circRNA_102481, the exosomes of the si-NC and si-circRNA_102481 cells were extracted and co-cultured with PC9/GR and HCC827/ER cells in our study. The results of MTT assay showed that, with gefitinib or erlotinib treatment, the cell viability of PC9/GR and HCC827/ER cells was significantly inhibited when they were co-cultured with exosomes of the si-circRNA_102481 group, and the IC50 value was also markedly decreased (IC50, 3.12 vs. 4.88 μmol/l and 5.85 vs. 12.04 μmol/l, respectively), compared with that of co-culture of exosomes in the si-NC group (Figure 6A, 6B; p<0.05).

In addition, after treatment with 2 μmol/l gefitinib or 4 μmol/l erlotinib for 24 h, the results of Annexin V/PI assay demonstrated that the apoptosis rate was markedly increased when cells were co-cultured with exosomes of the si-circRNA_102481 group compared with that of the corresponding si-NC group (Figure 6C, 6D; p<0.05). These results suggested that exosomal circRNA_102481 could inhibit EGFR-TKIs resistance NSCLC cell proliferation and promote cell apoptosis. It also indirectly indicated that circRNA_102481 mainly acts through exosomes.

### Effects of circRNA_102481 on EGFR-TKIs-sensitive NSCLC cells

**Figure 7:**
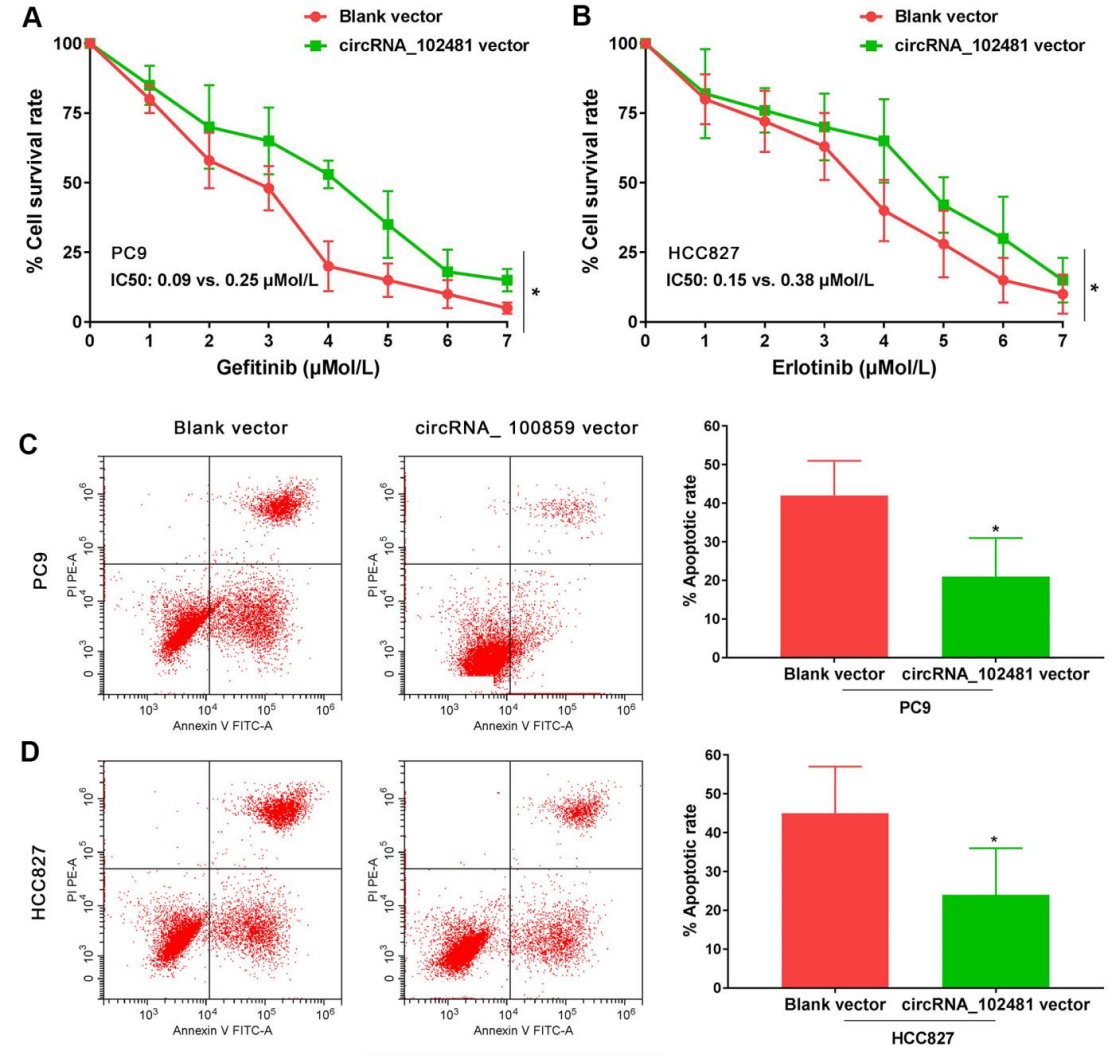
**Functional validation assay of exosomes circRNA_102481 on EGFR-TKIs sensitive cells *in vitro* (n=3).**(**A**, **B**) MTT assay showed the effects of circRNA_102481 on EGFR-TKIs sensitive cell proliferation. (**C**, **D**) Annexin V/PI assay showed the effects of circRNA_102481 on EGFR-TKIs sensitive cell apoptosis. circRNA_100859 vector: circRNA_100859 overexpression vector. *p<0.05 versus blank vector group.

In our study, the full length of circRNA_100859 vector and blank vector were transfected in PC9 and HCC827 cells. MTT assay revealed that the gefitinib (0.0, 0.025, 0.05, 0.1, 0.2, 0.4, 0.8 μmol/L) IC50 value in PC9 cells and the erlotinib (0.0, 0.025, 0.05, 0.1, 0.2, 0.4, 0.8 μmol/L) IC50 value in HCC827 cells were markedly increased in the circRNA_ 100859 vector group compared with those of the blank vector group (IC50, 0.09 vs. 0.25 μmol/l and 0.15 vs. 0.38 μmol/l, respectively; Figure 7A, 7B; p<0.05). In addition, after treatment with 0.1 μmol/l gefitinib or 0.2 μmol/l erlotinib for 24 h, the results of the Annexin V/PI assay demonstrated that the apoptosis rate was markedly decreased in the circRNA_100859 vector group compared with that of the blank vector group (Figure 7C, 7D; p<0.05). It’s demonstrated that circRNA_102481 overexpression could promote EGFR-TKIs sensitive NSCLC cell proliferation and inhibit cell apoptosis *in vitro*.

### CircRNA_102481 serves as a miR-30a-5p sponge to regulate ROR1 expression

**Figure 8:**
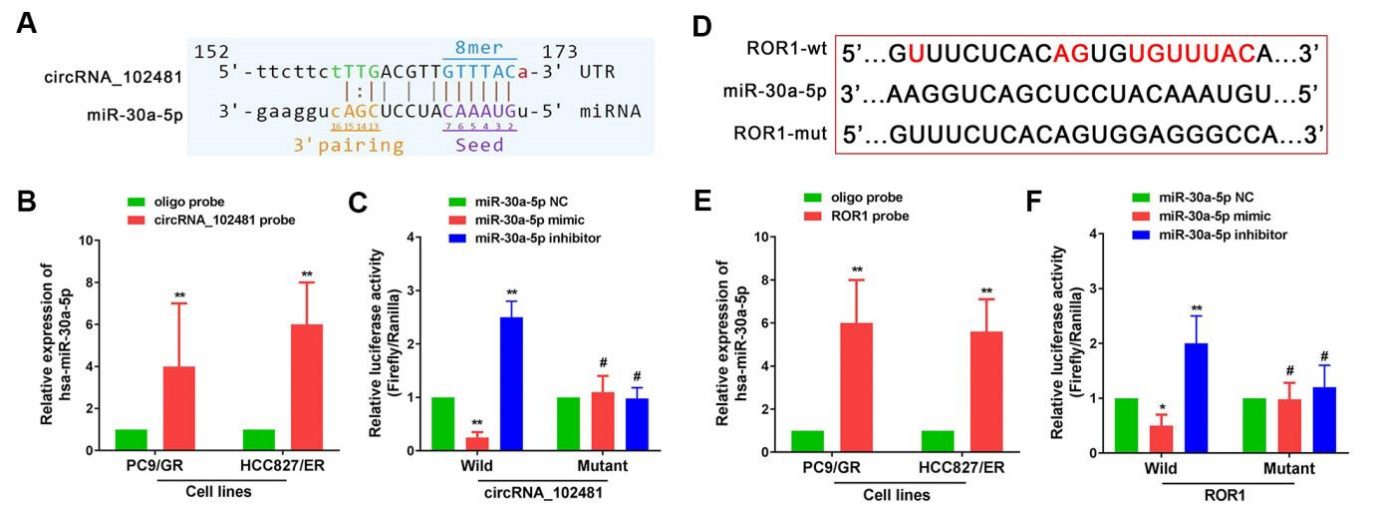
**circRNA_102481 serves as a miR-30a-5p sponge to regulate ROR1 expression.** The detailed potential binding sequences of circRNA_102481 to miR-30a-5p (**A**) and ROR1 to miR-30a-5p (**D**). miR-30a-5p of PC9/GR and HCC827/ER cells was abundantly pulled down by circRNA_102481 (**B**) or ROR1 (**E**). Dual-luciferase reporter assay confirmed that miR-30a-5p competitively targeted circRNA_102481(**C**), and that ROR1 is a direct target of miR-30a-5p (**F**). ^#^*p* > 0.05, *p< 0.05, **p<0.01.

It is well known that a circRNA can act as a miRNA sponge to regulate targeted gene expression. In our study, miR-30a-5p was predicted as the highest targeted miRNA for circRNA_102481, and ROR1 showed a relatively high target score in miR-30a-5p target gene prediction. Arraystar's proprietary software demonstrated that miR-30a-5p could specifically bind to circRNA_102481 (Figure 8A), and that ROR1 could specifically bind to miR-30a-5p (Figure 8D).

miR-30a-5p was extracted after circRNA_102481 or ROR1 pull-down assay, and RT-qPCR assay showed that miR-30a-5p of PC9/GR and HCC827/ER cells was abundantly pulled down by circRNA_102481 or ROR1 (Figure 8B, 8E; p<0.05). Dual-luciferase reporter assay confirmed that miR-30a-5p competitively targeted circRNA_102481, and that ROR1 is a direct target of miR-30a-5p (Figure 8C, 8F; p<0.05).

**Figure 9:**
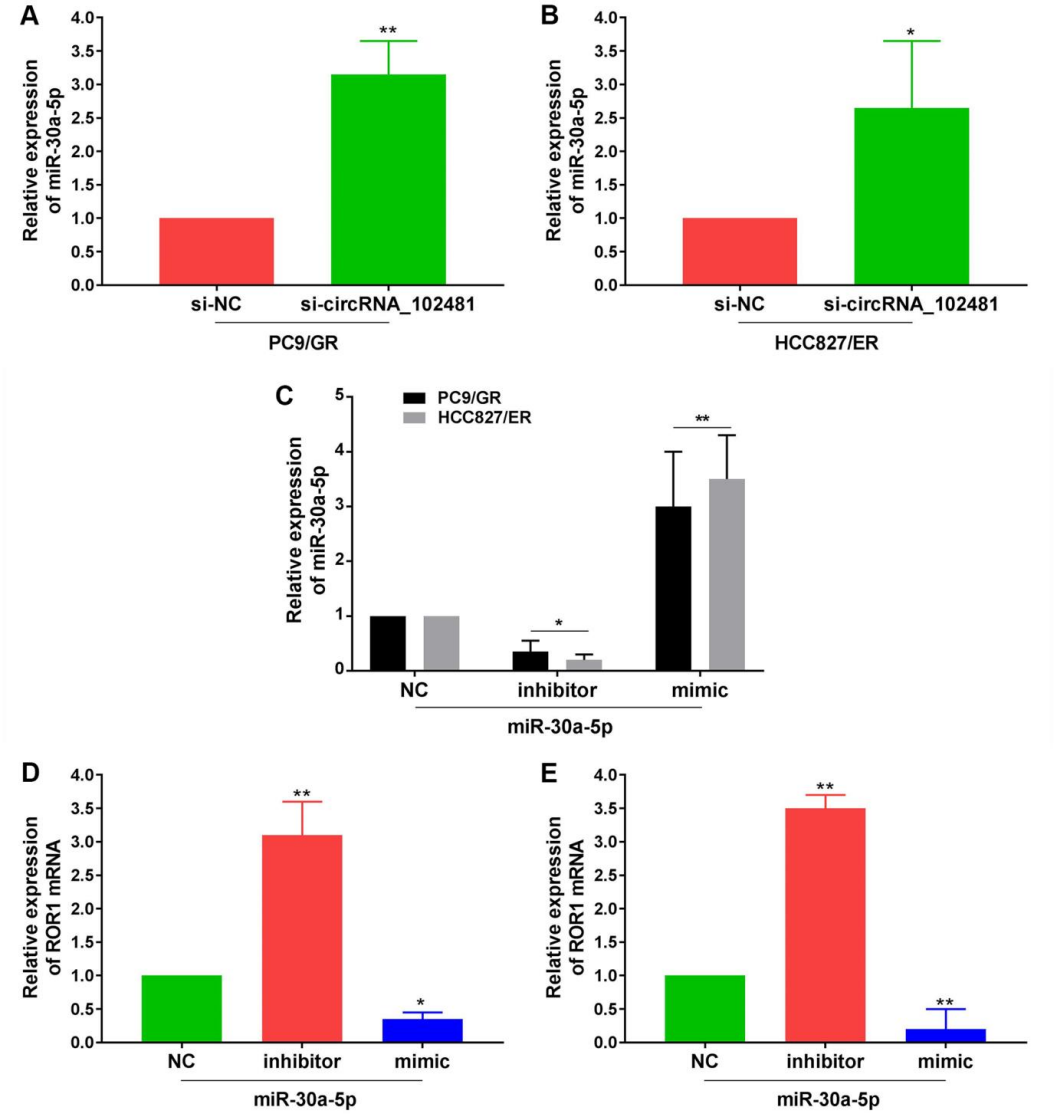
**Effects of exosomes on circRNA_102481-miR-30a-5p-ROR1 Axis.** (**A**, **B**) RT-qPCR assay showed miR-30a-5p expression in PC9/GR or HCC827/ER cells of si-NC and si-circRNA_102481 groups. (**C**) Transfection efficiency of miR-30a-5p mimic and miR-30a-5p inhibitor was verified by RT-qPCR assay. **p<0.01 versus miR-30a-5p NC group cells. (**D**, **E**) qRT-PCR demonstrated that ROR1 mRNA expression were significantly decreased in miR-30a-5p mimic group, and increased in miR-30a-5p inhibitor group. *p<0.05, **p<0.01 versus miR-30a-5p NC group.

In addition, RT-qPCR assay demonstrated that miR-30a-5p expression in PC9/GR and HCC827/ER cells was significantly increased in the si-circRNA_102481 group compared with that in the si-NC group (Figure 9A, 9B; p<0.05). Furthermore, miR-30a-5p NC, miR-30a-5p mimic and miR-30a-5p inhibitor were successfully transfected into PC9/GR and HCC827/ER cells (Figure 9C; p<0.05). RT-qPCR assay demonstrated that ROR1 mRNA expression was significantly increased in the miR-30a-5p inhibitor group and decreased in the miR-30a-5p mimic group compared with its expression in the miR-30a-5p NC group (Figure 9D, 9E; p<0.05).

### Effects of exosomes on the circRNA_1024810/miR-30a-5p/ROR1 axis

**Figure 10:**
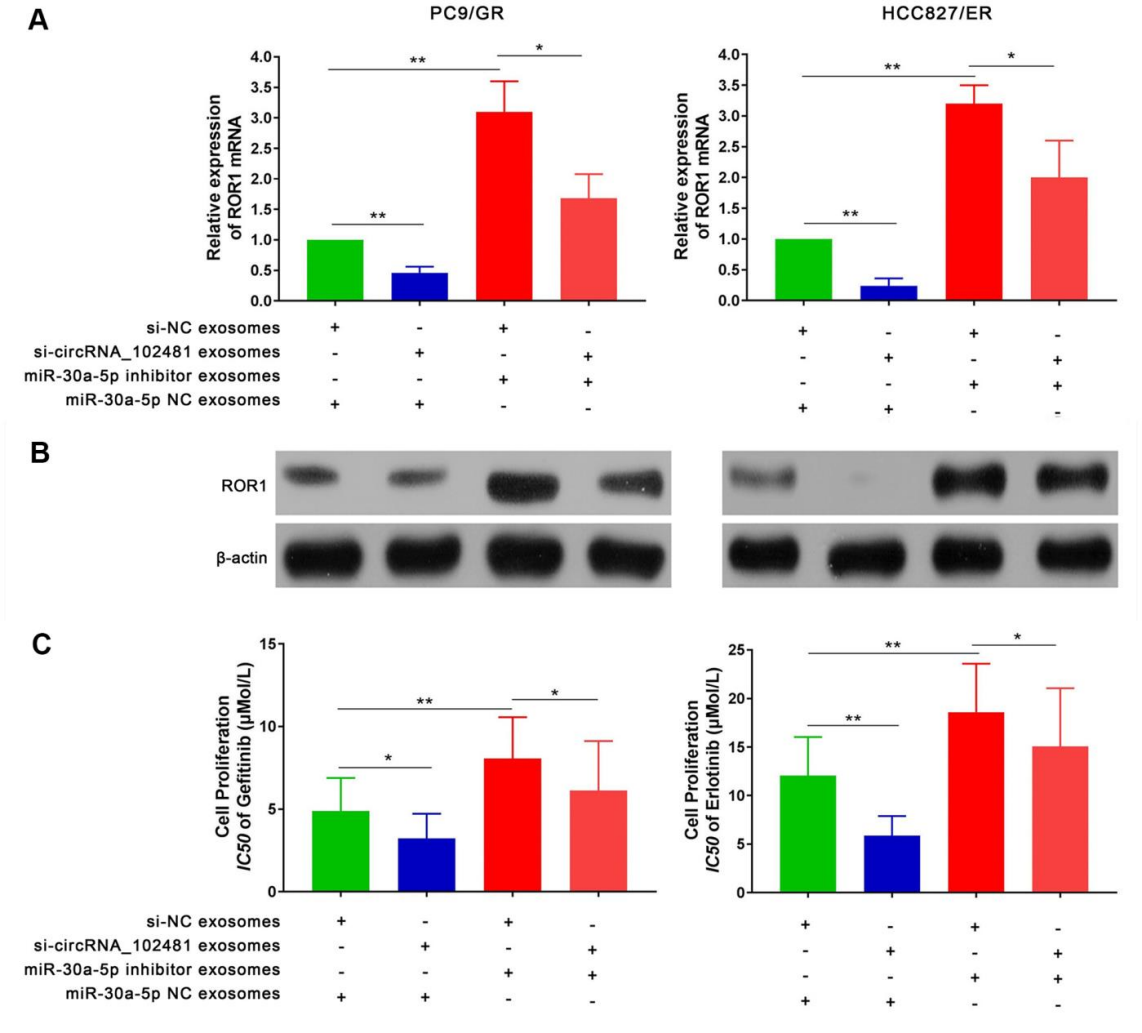
**Effects of exosomes on circRNA_102481-miR-30a-5p-ROR1 Axis.** (**A**, **B**) Relative ROR1 mRNA and protein were analyzed in PC9/GR and HCC827/ER cells the cells were co-cultured with exosomes of the si-NC, si-circRNA_102481, and miR-30a-5p NC, miR-30a-5p inhibitor using RT-qPCR and western blot assay, respectively. (**C**) IC50 of gefitinib or erlotinib the cells were co-cultured with exosomes of the si-NC, si-circRNA_102481, and miR-30a-5p NC, miR-30a-5p inhibitor was detected by MTT assay. *p< 0.05, **p<0.01.

To further explore the interaction between exosomes and the circRNA_ 1024810/miR-30a-5p /ROR1 axis, a series of experiments were performed. First, RT-qPCR and western blot assays demonstrated that the ROR1 mRNA and protein levels in PC9/GR and HCC827/ER cells were significantly decreased when the cells were co-cultured with exosomes of the si-circRNA_102481 group. By contrast, exosomes of the miR-30a-5p inhibitor group increased ROR1 mRNA and protein expression, while co-culture with exosomes of the si-circRNA_102481 and miR-30a-5p inhibitor groups could reverse these effects (Figure 10A, 10B; p<0.05). MTT assay showed that the IC50 of gefitinib or erlotinib was significantly decreased in PC9/GR cells or HCC827/ER cells when co-cultured with exosomes of the si-circRNA_102481 groups, whereas exosomes of the miR-30a-5p inhibitor group increased the IC50 of gefitinib or erlotinib. Similarly, co-culture with exosomes of the si-circRNA_102481 and miR-30a-5p inhibitor groups could counteract these effects (IC50, 4.88 vs. 3.12 vs. 8.07 vs. 6.12 μmol/l and 12.04 vs. 5.85 vs.18.58 vs. 15.06 μmol/l, respectively; Figure 10C; p<0.05).

### Large sample validation and correlation analysis

**Figure 11:**
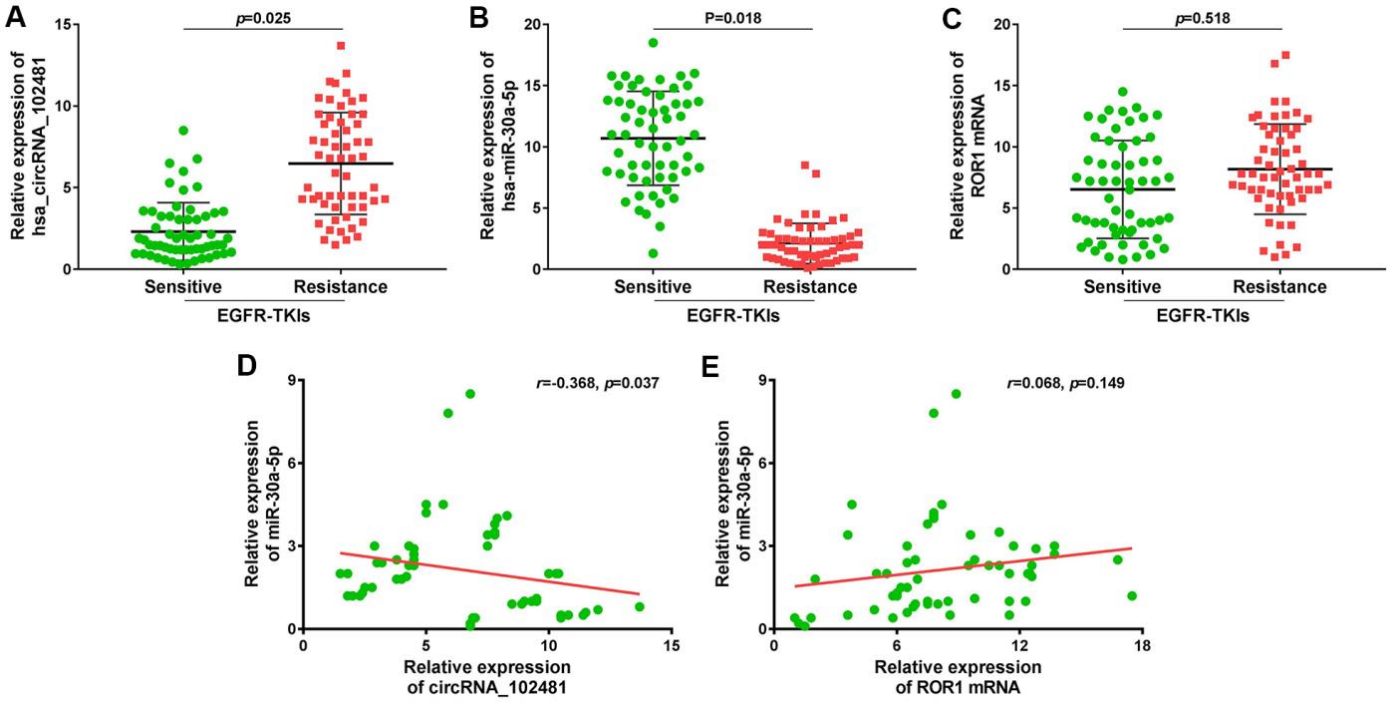
**Large sample validation and correlation analysis.** (**A**–**C**) RT-qPCR analysis of exosomes circRNA_102481, exosomes miR-30a-5p, exosomes ROR1 mRNA before and after EGFR-TKIs resistance in 58 NSCLC patients. (**D**, **E**) Pearson’s correlation analysis of between exosomes circRNA_102481 and exosomes miR-30a-5p, between exosomes miR-30a-5p and exosomes ROR1 mRNA.

A total of 58 patients with NSCLC treated with EGFR-TKIs (gefitinib or erlotinib) were enrolled in our study. The serum exosomes of these 58 patients were extracted before and after developing EGFR-TKIs resistance. RT-qPCR revealed that exosomes containing circRNA_102481 were evidently up-regulated (Figure 11A; p=0.025), whereas exosomes containing miR-30a-5p were significantly down-regulated (Figure 11B; p=0.018) in patients with EGFR-TKIs resistance (n=58), compared with the numbers of matched exosomes in EGFR-TKIs-sensitive patients (n=58). However, there were no differences in ROR1 mRNA expression in exosomes before and after developing EGFR-TKIs resistance in patients with NSCLC (Figure 11C; p=0.518). In addition, Pearson’s correlation analysis showed that circRNA_102481 expression in exosomes was significantly negatively correlated with miR-30a-5p expression in exosomes (Figure 11D; r=-0.368; p=0.037), but there was no significant linear correlation between miR-30a-5p expression in exosomes and ROR1 mRNA expression in exosomes (Figure 11E; r=0.068; p=0.149).

### ROR1 expression in cells and exosomes

**Figure 12:**
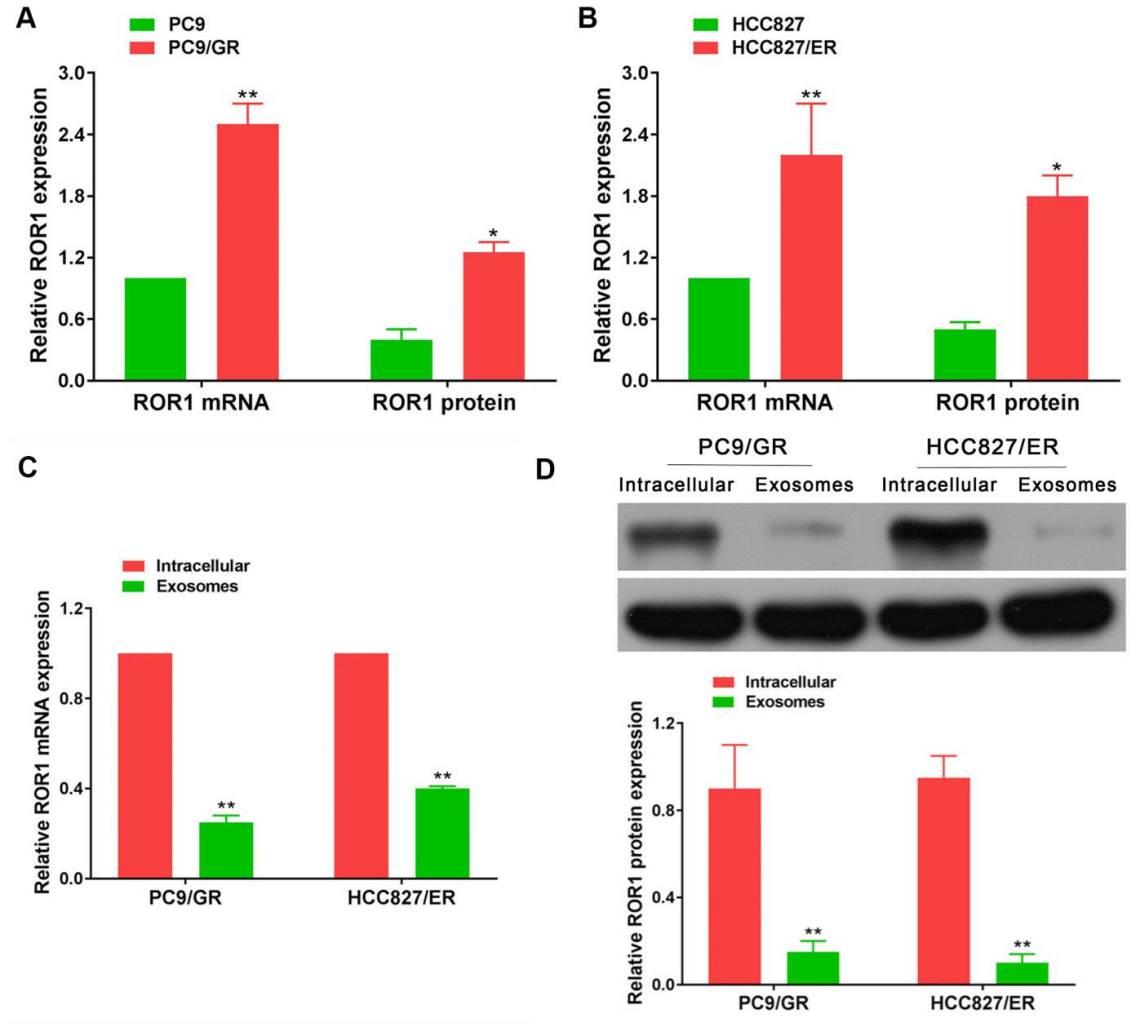
**ROR1 expression in cells and exosomes.** (**A**, **B**) ROR1 mRNA and protein levels were significantly up-regulated in EGFR-TKIs resistant cells (PC9/GR and HCC827/ER), *p<0.05, **p<0.01 versus matched sensitive cells (PC9 and HCC827). (**C**, **D**) ROR1 mRNA and protein expression levels in exosomes were significantly lower than those in cells. *p<0.05, **p<0.01 versus cells.

In our large sample validation results, there were no differences in ROR1 mRNA expression in exosomes before and after developing EGFR-TKIs resistance in patients with NSCLC. To further explore the levels of ROR1 expression in EGFR-TKIs-resistant cells, ROR1 mRNA and protein detection was performed. It was found that ROR1 mRNA and protein levels were significantly up-regulated in EGFR-TKIs resistant cells (PC9/GR and HCC827/ER) compared with its expression level in sensitive cells (PC9 and HCC827) (Figure 12A, 12B; p<0.05). However, in PC9/GR and HCC827/ER cells, the ROR1 mRNA and protein expression levels in exosomes were significantly lower than those in cells (Figure 12C, 12D; p<0.05), indicating that ROR1 mRNA and protein is not secreted in the form of exosomes by PC9/GR and HCC827/ER cells to the tumor microenvironment.

### Association between the exosomes circRNA_1024810/miR-30a-5p/ROR1 axis and clinicopathological variables

**Figure 13:**
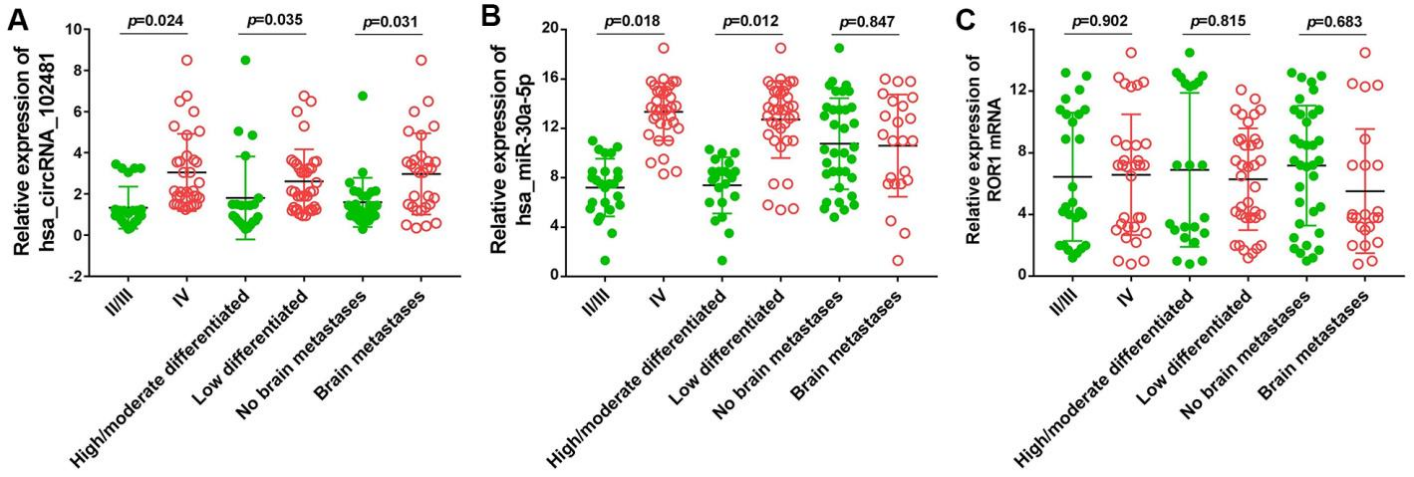
**Relationship between exosomes circRNA_102481-miR-30a-5p-ROR1 axis with clinicopathological variables.** (**A**) exosomes circRNA_102481, (**B**) exosomes miR-30a-5p, (**C**) exosomes ROR1 mRNA.

The association between the exosomes circRNA_1024810/miR-30a-5p/ROR1 axis and clinicopathological variables was analyzed by Fisher’s exact and χ2 tests. The OS durations in the different groups were compared using the Kaplan-Meier method and the log-rank test. At baseline, Fisher’s exact and χ2 tests demonstrated that circRNA_102481 expression in exosomes was positively associated with TNM stage and negatively associated with tumor differentiation status (Figure 13A; p<0.05), whereas, miR-30a-5p expression in exosomes was negatively associated with TNM stage and positively associated with tumor differentiation status (Figure 13B; p<0.05). However, the status of ROR1 mRNA expression in exosomes showed no correlation with TNM stage or tumor differentiation status (Figure 13C; p>0.05). Notably, aberrantly high expression of circRNA_1024810 in exosomes was closely associated with brain metastasis (Figure 13A; P<0.05), while miR-30a-5p (Figure 13B; p>0.05) and ROR1 mRNA expression (Figure 13C; p>0.05) in exosomes did not correlate with brain metastasis.

**Figure 14:**
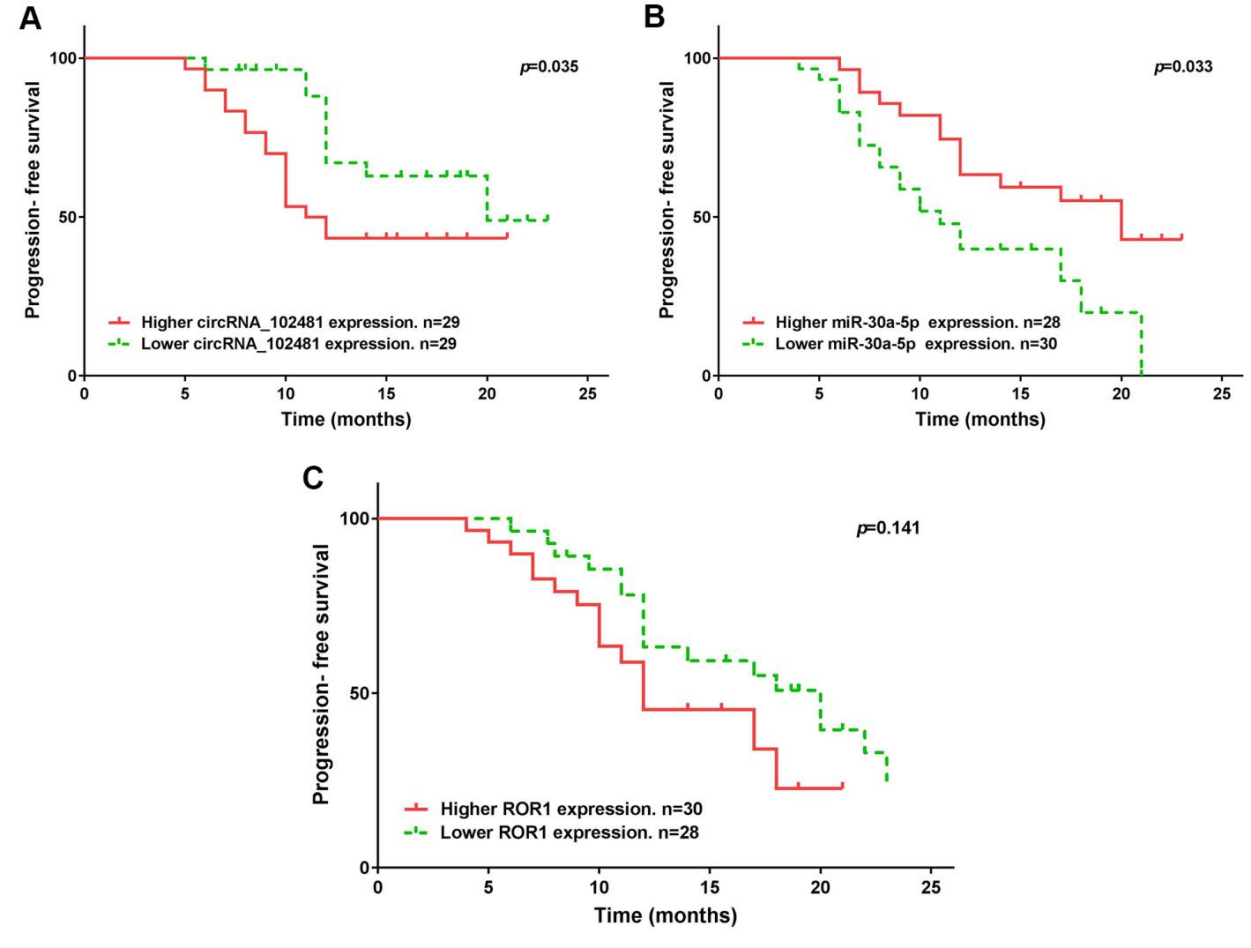
**Kaplan-Meier PFS curve stratified by exosomes circRNA_102481- miR-30a-5p-ROR1 axis expression.** PFS duration between higher and lower (**A**) exosomes circRNA_102481, (**B**) exosomes miR-30a-5p, (**C**) exosomes ROR1 mRNA.

**Figure 15:**
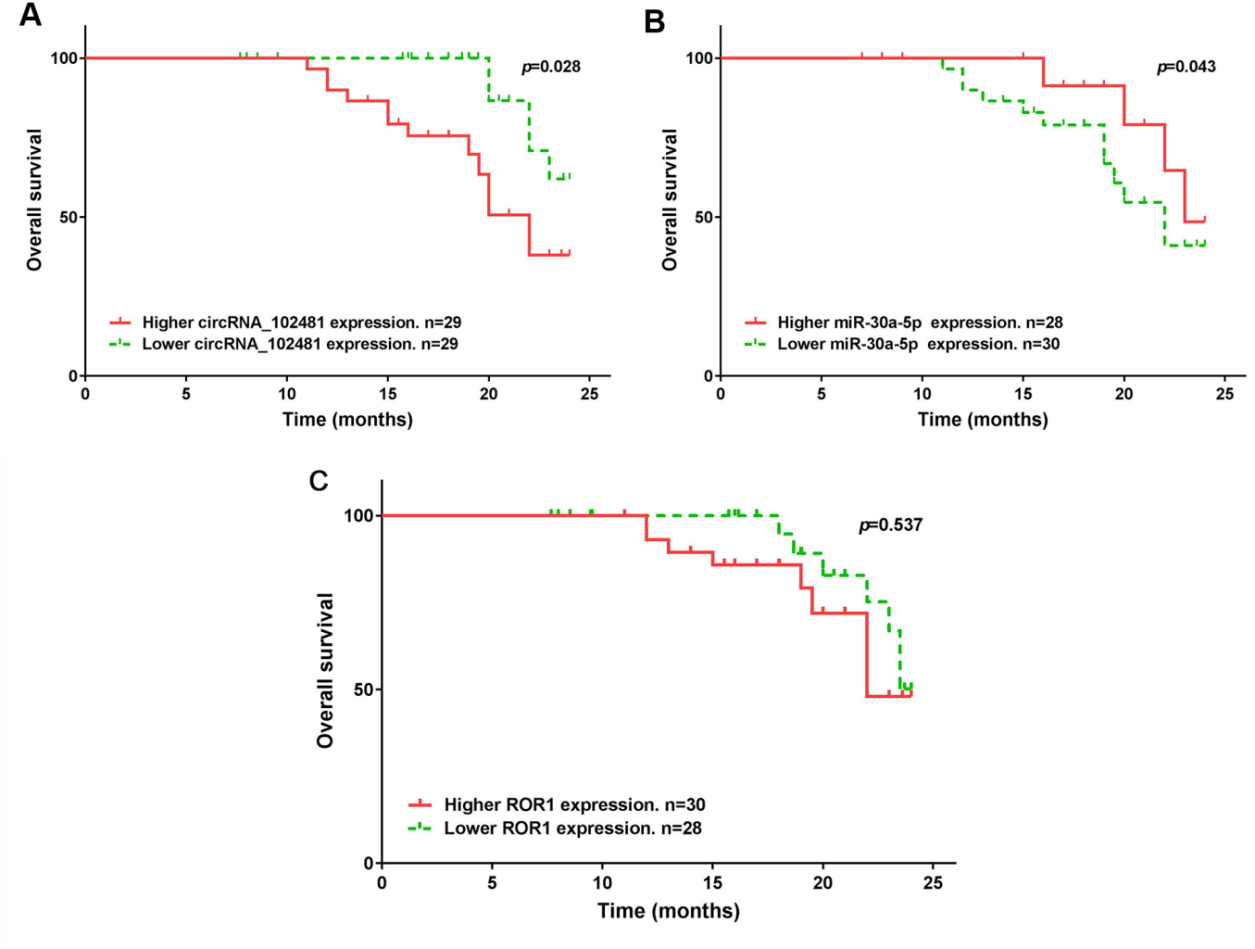
**Kaplan-Meier OS curve stratified by exosomes circRNA_102481- miR-30a-5p-ROR1 axis expression.** OS duration between higher and lower (**A**) exosomes circRNA_102481, (**B**) exosomes miR-30a-5p, (**C**) exosomes ROR1 mRNA.

The median expression of the circRNA_1024810/miR-30a-5p/ROR1 axis was set as the cutoff value. The results of Kaplan-Meier method and log-rank test showed that patients with higher circRNA_1024810 expression in exosomes had shorter PFS (Figure 14A; p=0.035) and OS (Figure 15A; p=0.028) times, whereas patients with higher miR-30a-5p expression in exosomes had better PFS (Figure 14B; p=0.033) and OS (Figure 15B; p=0.043) times. However, the PFS and OS duration did not differ between patients with higher and lower ROR1 mRNA expression in exosomes (Figure 14C, 15C, respectively; both p>0.05).

## DISCUSSION

Exosomes are an important part of the extracellular environment. Studies [22] have shown that there are about 2×10^15^ exosomes in the normal human circulatory system, while there are up to 4×10^15^ in tumor patients, and their heterogeneity can increase with tumor progression. A growing number of studies have shown that exosomes involved in the initiation and progression of a variety of tumors. Yu et al. [23] reported that exosomes-delivered nucleic acid and proteins partly facilitate the tumorigenesis, metastasis and resistance of breast cancer. Nedaeinia et al. [24] demonstrated that circulating exosomes were novel biomarkers for gastrointestinal cancer. Chen et al. [25] found that Exosomes participation in lung cancer initiation, progression and metastasis as well as being involved in angiogenesis, epithelial-mesenchymal transition (EMT), immune escape, and drug resistance.

Exosomes contain biological macromolecules, and the function of exosomes is to transport this biological information to neighboring cells and play essential roles in intercellular communication and signal transduction. Current studies [26–29] have shown that exosomal circRNA hold great promise as novel biomarkers for clinical diagnosis. Dai et al. [30] reported that exosomal circRNA_100284 involved in the malignant transformation of human hepatic cells by accelerating the cell cycle and promoting cell proliferation. Zhang et al. [31] found that exosomal circRNA derived from gastric tumor promotes white adipose browning by targeting the miR-133/ PRDM16 pathway. Li et al. [32] demonstrated that exosomal circ_IARS secreted by pancreatic cancer cells regulates endothelial monolayer permeability to promote tumor metastasis. Shang et al. [33] showed that exosomal circ_PACRGL promotes progression of colorectal cancer via the miR-142-3p/miR-506-3p-TGF-β1 axis. However, the role of tumor-derived exosomal circRNA on EGFR-TKIs resistance still remains unclear yet.

In the present study, serum exosomes were isolated from five patients with NSCLC, and differentially expressed circRNAs were identified via circRNA microarray for the first time. Our results revealed that circRNA_102481 was significantly up-regulated in serum exosomes of EGFR-TKIs-resistant patients, which was further validated by RT-qPCR assay. In addition, *in vitro* functional validation assays found that circRNA_102481 silencing inhibited EGFR-TKIs-resistant NSCLC cell proliferation and promoted apoptosis, circRNA_102481 overexpression could promoted EGFR-TKIs sensitive NSCLC cell proliferation and inhibited cell apoptosis which suggested that circRNA_102481 can contribute to EGFR-TKIs resistance in NSCLC.

Exosomes can derive from various types of cells. The present study investigated whether circRNA_102481 derives from exosomes secreted by tumor cells. Our results revealed that circRNA_102481 was up-regulated in EGFR-TKIs-resistant cells and exosomes compared with its expression in sensitive cells and exosomes. In addition, it was found that circRNA_102481 expression levels in exosomes were almost equal to those in CCM, and that circRNA_102481 in CCM was little influenced by treatment with RNase alone but significantly decreased following treatment with RNase and Triton X-100. Furthermore, knockdown of circRNA_102481 in EGFR-TKIs-resistant NSCLC cells caused a significant decrease in circRNA_102481 levels in exosomes. These results suggested that circRNA_102481 can be secreted by EGFR-TKIs resistant NSCLC cells, and that exosomes are the main carriers of extracellular circRNA_102481. Further functional validation assays demonstrated that si-circRNA_102481 transported by exosomes can inhibit cell proliferation and promote cell apoptosis, similarly to circRNA_102481 knockdown.

Previous studies [34, 35] have demonstrated that the main function of circRNA is acting as a miRNA sponge by competitively binding to its target miRNA, thereby regulating the expression of target genes. In our study, the interaction between miR-30a-5p and circRNA_102481 or ROR1 was predicted by starBase software and confirmed by RNA pull-down and dual-luciferase reporter assays. Rescue assay validated that ROR1 mRNA and protein levels were positively regulated by exosome-derived circRNA_102481, but these effects could be reversed by exosome-derived miR-30a-5p. This indicated that exosome-derived circRNA_102481 can regulate ROR1 expression by sponging miR-30a-5p. In addition, large-sample validation (n=58) found that exosome-derived circRNA_102481 was significantly up-regulated, while exosome-derived miR-30a-5p was down-regulated in EGFR-TKIs-resistant patients. Furthermore, the expression of circRNA_102481 and miR-30a-5p in exosomes was closely associated with TNM stage, tumor differentiation status, and PSF and OS duration.

CircRNA_102481 is an exonic circRNA (NM_014173) located in chromosome 19:17278895 (GRCh38.p12), and its host gene is BABAM1. To the best of our knowledge, this is the first report about circRNA_102481 on cancer regulation. MiR-30a-5p, a member of the miR-30 family with 22 bp in length, is highly conserved in humans [36]. MiR-30a-5p mainly plays a tumor suppressor role in various cancer types due to its alleviation of cell malignant phenotypes [37, 38]. Li et al. [39] found that miR-30a-5p inhibits breast cancer cell proliferation, migration and invasion by regulating the ERK/ETS-1 signaling pathway. Zhou et al. [40] reported that down-regulation of miR-30a-5p is associated with poor prognosis and promotes chemoresistance to gemcitabine in pancreatic ductal adenocarcinoma. Świtlik et al. [41] reported that miR-30a-5p may serve as a promising biomarker for NSCLC. Zhang et al. [42] showed that miR-30a-5p promotes cholangiocarcinoma cell proliferation through targeting SOCS3. In our study, miR-30a-5p was demonstrated to be involved in EGFR-TKIs resistance. Notably, no differences in ROR1 mRNA expression were observed in exosomes before and after developing EGFR-TKIs resistance in large-sample validation. Our cell experiments also showed the ROR1 mRNA and protein expression was significantly up-regulated in EGFR-TKIs-resistant cells, but its expression level in exosomes was significantly lower than that in cells, which indicated that ROR1 mRNA and protein are not secreted in the form of exosomes by EGFR-TKIs-resistant cells to the tumor microenvironment. ROR1 mainly regulates the occurrence and development of tumors in cells. RTK-like orphan receptor 1 (ROR1) is an evolutionarily conserved, type-I membrane protein that is expressed during embryogenesis [43]. Overexpression of ROR1 is known to be closely associated with tumor growth, metastasis and chemotherapy resistance in several human tumor types [44–46]. Our results suggested that ROR1 may be involved in EGFR-TKIs resistance, but its exact function needs further exploration.

However, there were several limitations in our study. Firstly and most critically, exosomes can derive from various cell types. In our study, PC9/GR and HCC827/ER cells were employed, and it was demonstrated that resistant cells can secrete exosomes containing circRNA_102481 to promote EGFR-TKIs resistance, but could not demonstrate whether exosome-derived circRNA_102481 can be secreted by other cells in the body simultaneously. Secondly, our study only focused on cell function *in vitro*, whereas it lacked animal experiments for further *in vivo* verification. Thirdly, only 58 patients were enrolled in our study; thus, the sample size was relatively small, and the longest follow-up time was 25 months, which may ultimately affect the judgment of OS.

## CONCLUSIONS

In conclusion, our study revealed for the first time that exosomal circRNA_102481 was significantly up-regulated in NSCLC with EGFR-TKIs resistance. Tumor-derived exosomal circRNA_102481 could contribute to EGFR-TKIs resistance in NSCLC by sponging miR-30a-5p to enhance ROR1 expression. Exosomal circRNA_102481 may serve as a novel diagnostic biomarker and a therapeutic target for EGFR-TKIs resistance in NSCLC.

### Data availability

The datasets used/or analyzed during the current study are available from the corresponding author on reasonable request.

## Supplementary Material

Supplementary Figure 1

Supplementary Tables
