# Supplementary material for: Tumor-derived exosomal circRNA_102481 contributes to EGFR-TKIs resistance via the miR-30a-5p/ROR1 axis in non-small cell lung cancer
Source: Aging (Albany NY). 2021 May 5;13(9):13264–86. doi: 10.18632/aging.203011 (PMC8148492; doi:10.18632/aging.203011)
Supplement: Supplementary Figure 1 [file aging-13-203011-s001.pdf]

SUPPLEMENTARY FIGURE

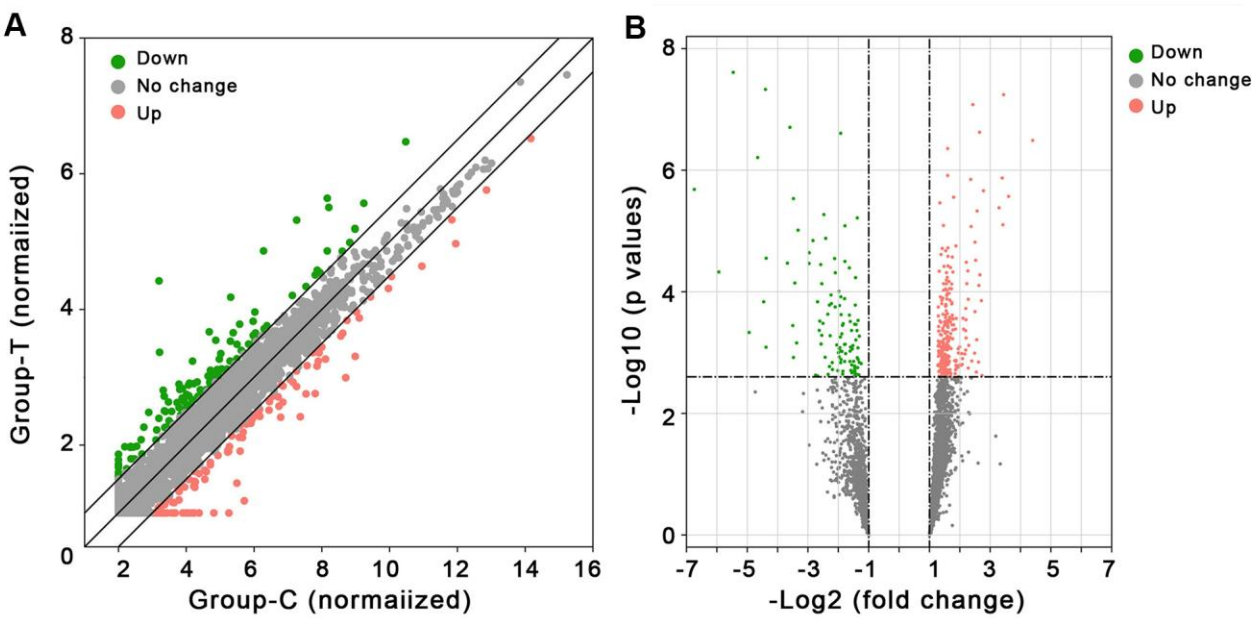

Supplementary Figure 1. Exosomal circRNA expression profile was established successfully. (**A**) Volcano plots were constructed using fold change values and P-values. The values of x and y axes in the scatter plot are the normalized signals of the samples (log2 scaled). (**B**) Scatter plot was for assessing the variation of circRNA expression. The vertical lines respectively correspond to 2.0-fold up and down, and the horizontal line represents a P-value of 0.05. The red point in the plot represents the up-regulated expressed circRNAs with statistical significance; the blue point in the plot represents the down-regulated expressed circRNAs with statistical significance.
