# Supplementary material for: Tumor-derived exosomal circRNA_102481 contributes to EGFR-TKIs resistance via the miR-30a-5p/ROR1 axis in non-small cell lung cancer
Source: Aging (Albany NY). 2021 May 5;13(9):13264–86. doi: 10.18632/aging.203011 (PMC8148492; doi:10.18632/aging.203011)
Supplement: Supplementary Tables [file aging-13-203011-s002.pdf]

SUPPLEMENTARY TABLES

Supplementary Table 1. The clinicopathologic characteristics of enrolled patients for circRNA CHIP.

	Age	Gender	Pathological types	TNM stage	Pathological stage
Patient 1	67	Female	Squamous cell carcinoma	IV	Low
Patient 2	54	Male	Squamous cell carcinoma	III	middle
Patient 3	60	Male	Squamous cell carcinoma	IV	Low
Patient 4	68	Female	Squamous cell carcinoma	IV	middle
Patient 5	59	Female	Squamous cell carcinoma	IV	Low

Supplementary Table 2. Patients'clinicopathologic characteristics (N=58).

Characteristics	Cases (n)
Age (Median /years)	65(42-75) y
Gender	
Male	38(65.52)
Female	20(34.48)
Pathological types	
Adenocarcinoma	0(0.00)
Squamous cell carcinoma	58(100.00)
TNM stage	
II/ III	25(43.11)
IV	33(56.89)
Pathological stage	
High and middle	22(37.93)
Low	36(52.07)
EGFR mutation site	
19del/L858R	51(87.93)
others	7(12.07)
